# Evidence of Reproductive Stress in Titanosaurian Sauropods Triggered by an Increase in Ecological Competition

**DOI:** 10.1038/s41598-017-14255-6

**Published:** 2017-10-23

**Authors:** Albert G. Sellés, Bernat Vila, Àngel Galobart

**Affiliations:** 1 Department of Mesozoic Faunas, Institut Català de Paleontologia “Miquel Crusafont”, c/Escola Industrial 23, 08201 Sabadell, Spain; 2 Museu Conca Dellà, c/del Museu 4, 25650 Isona, Spain

## Abstract

The occurrence of dinosaur pathologic eggs in the Late Cretaceous of Europe is well known, but their origin remains unclear. Here we expose the results of a detailed sampling of the conspicuous fossil record of Late Cretaceous titanosaurian eggs (oogenus *Megaloolithius*) from several southwestern Europe basins. After examining more than 450 samples, we observed a remarkable and statistically supported occurrence of multiple pathologic eggs in a relatively short stratigraphic range at the end of the early Maastrichtian, circa 71-70 Ma. All pathologic specimens exhibit multi-layered eggshell condition, a characteristic related to dystocia, or egg retention within the female uterus for an abnormal prolonged period of time. After exploring various scenarios, the occurrence of pathologic eggs is strongly correlated with an intense dinosaur faunal replacement that occurred during the early Maastrichtian in the Ibero-Armorican Island. Given that inter-species competiveness is proved to produce major affects in ecological communities, our results suggest that pathologies in the eggs of European titanosaurians could be a consequence of an increase in reproductive stress triggered by direct ecological competition between different dinosaurs. Thus, the present study provides a new perspective of how dinosaurs might have been affected by ecological/environmental disturbance.

## Introduction

To understand how species are affected by environmental disturbance is a crucial topic in both modern ecology and palaeoecology. Several studies demonstrate that environmental/ecological alterations force animal communities to restructure resource partitioning^1–5^. However, this reorganization is just one of the many consequences affecting the community when experiencing a new ecologic stability. Environmental changes and biotic disturbances can also drive ecological communities into stressed conditions, producing several negative effects upon organisms^6^. For instance, under unfavourable conditions oviparous amniotes may suffer an increase in reproductive stress that can be physically expressed in the malfunction of the reproductive system^7–10^.

**Figure 1:**
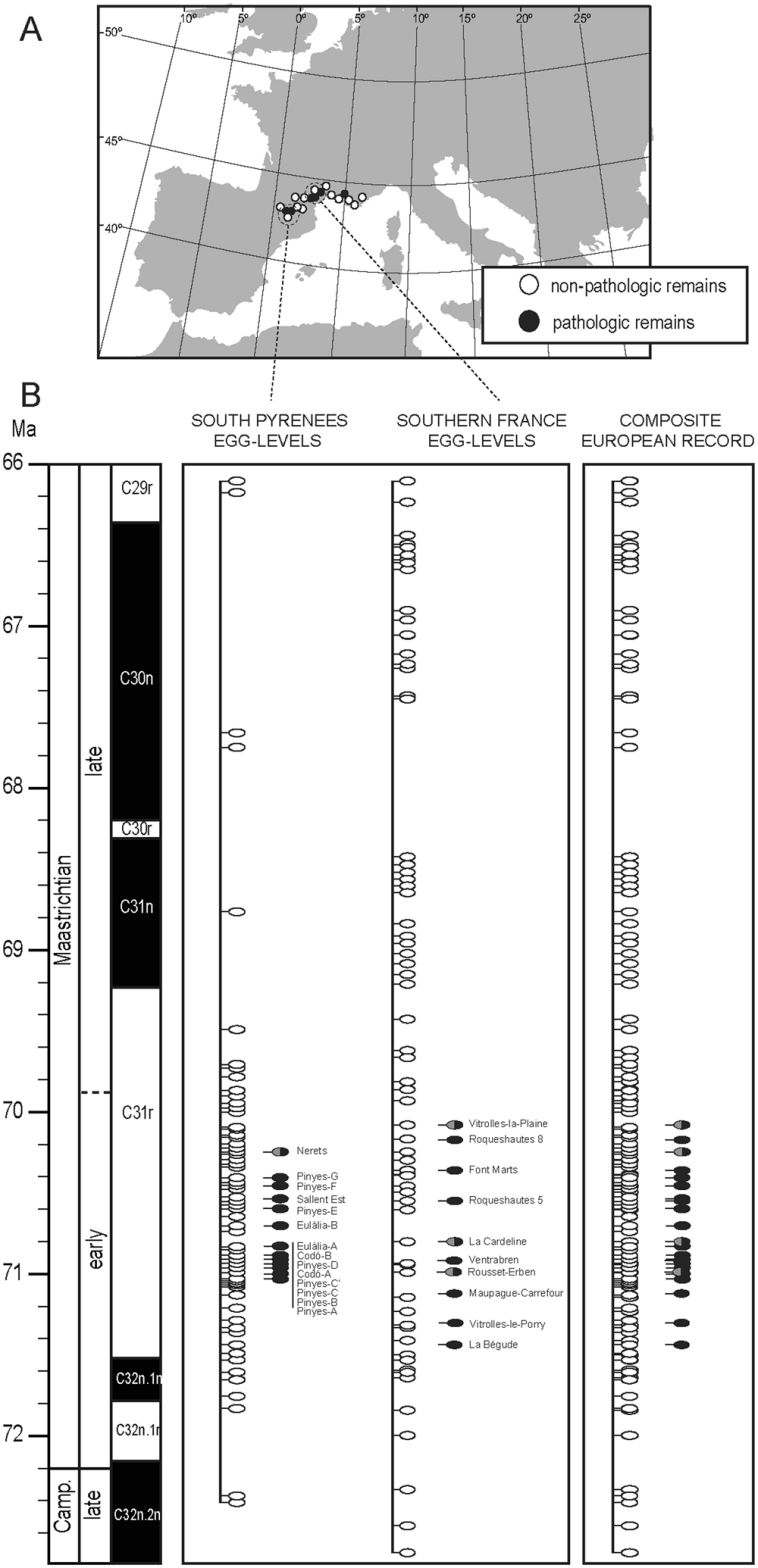
Distribution of titanosaurian normal and pathologic eggs in Europe. Geographical (**A**) and chronostratigraphical (**B**) distribution of the latest Cretaceous pathologic megaloolithid eggs by regions (southern Pyrenees and southern France) and as a composite records. Pathologic remains identification: solid black spots represent those remains belonging to the oospecies *Megaloolithus siruguei*; half gray-half black spots belong to *Megaloolithus mamillare*. Geographic map modified from http://www.freeworldmaps.net/ using Adobe Illustrator CC 2015.2.1. Data source^30–33,48,50,81,82,84^. See Figs S1 and S2 for further details.

The fossil record offers several examples of pathologic oological remains, especially in dinosaurs^9–14^, but very few convincing hypotheses are proposed to explain their origin. In this regard, the Upper Cretaceous formations of southwestern Europe offer an exceptional continuous record of pathologic megaloolithid egg^14–17^ (but see^18^; Fig. 1, Supplementary Information), an egg type attributed to titanosaurian sauropods^19–21^. This framework provides a unique opportunity to explore how dinosaur faunas could physiologically have responded to ecological/environmental perturbations.

Historically, the presence of dinosaur pathologic eggs has been linked with climatic changes^22–25^, or considered as one of the causes of the demise of dinosaurs^15^. Nevertheless, the former hypothesis has never been explored in detail, and the latter one has been widely rejected^9,13,16,17,26,27^. Here, we explore several scenarios to address the possible trigger event that favoured the overproduction of pathologic eggs in titanosaurians from Europe. In order to solve this question, the stratigraphic distribution of the pathologic eggs is firstly evaluated from a statistic perspective. Given that isotopic signature of eggshells can be used as a proxy for inferring change in both environmental and biological condition^15^, stable isotopes (δ^13^C and δ^18^O) of both normal and pathologic eggshells are measured and compared. Finally, these data are evaluated under different ecologic scenarios that occurred at the end of the Cretaceous (Maastrichtian time span) in southwestern Europe. As a result, we provide substantial evidence that abnormal eggs were the consequence of an intense ecological perturbation.

## Results

### Stratigraphic distribution and statistical significance of pathologic eggs

In southwestern Europe, titanosaurian eggs occur in non-marine deposits ranging from the upper Campanian to the uppermost Maastrichtian^28–33^. After sampling more than 90 consecutive stratigraphic egg-levels in 22 continuous and composite stratigraphic sections of the southern Pyrenees (Catalonia), we documented the occurrence of several *in situ* abnormal eggs in 14 egg-horizons (see Fig. 1; see also Supplementary Information).

Taking the fossil egg record as a whole, pathologic eggshells are very scarce at the region (0.5–2.5%)^16^, but most of them occur in a relatively short stratigraphic range in the lower Maastrichtian; thereby representing 9.3% of the fossil egg record in that interval (Fig. 1B; see also Supplementary Table S1). According to poultry industry data, under controlled non-stressed conditions, pathologic eggs barely represent the 2% of the total production^34,35^. These values are much lower than that of the studied region, where pathologic remains represent about 9.3% of the record.

**Figure 4:**
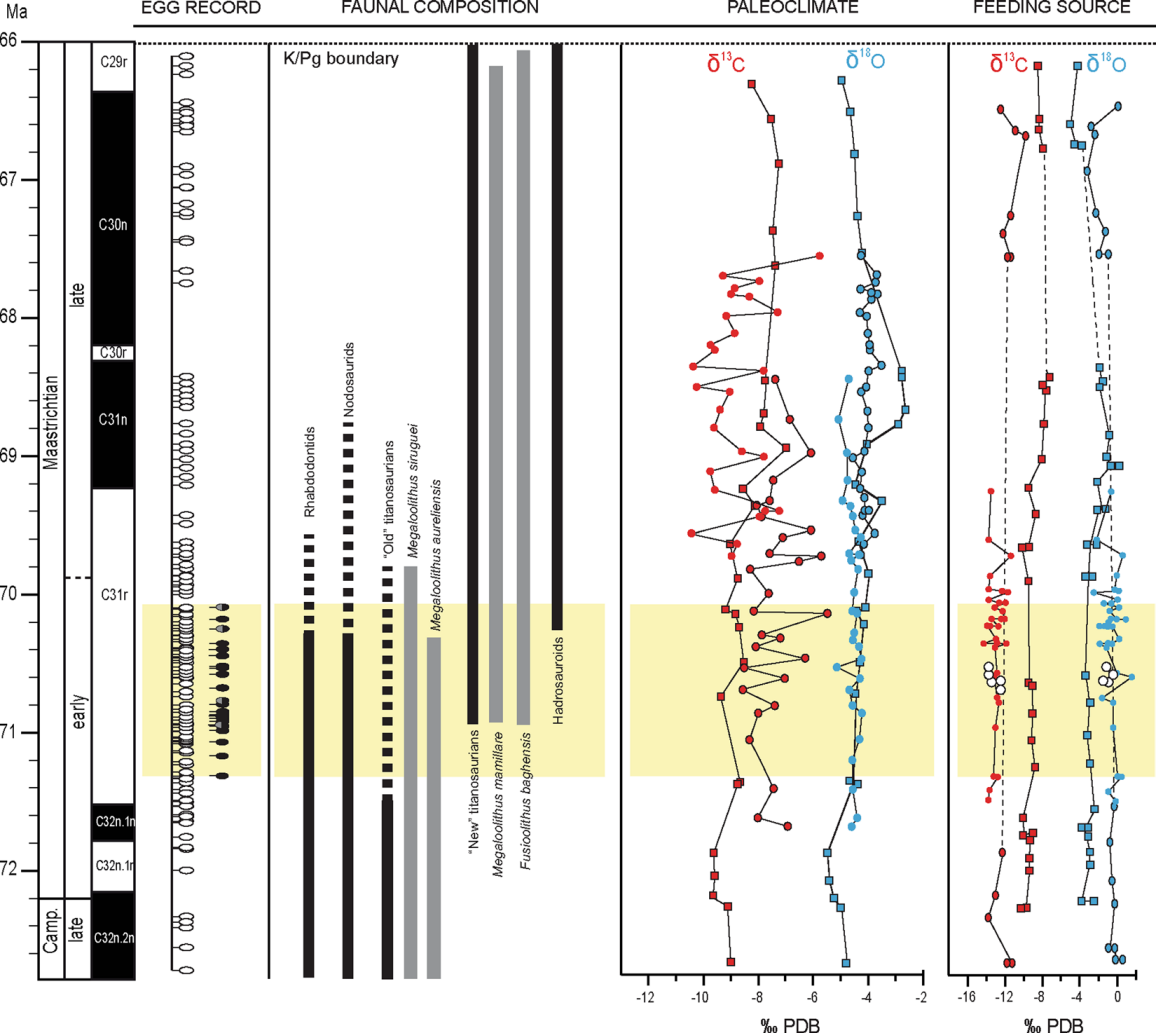
Testing environmental/biotic perturbation hypotheses. Correlation between normal (white marks) and pathologic (black marks) egg remains in southwestern Europe and the three tested hypotheses. From left to right: (1) latest Cretaceous European dinosaur faunal turnover, (2) stable isotope signal of pedogenic carbonate as climate proxy, (2) stable isotope signal of normal and pathologic (open circles) titanosaurian eggshells as food resource proxy. Open red circles indicate data from southern Pyrenees, close red circles from Provence, and closed Square from northern Pyrenees. Isotope data source^48,50^. Faunal occurrence source^31–33,71,84^.

All the pathologic eggs and eggshells recovered from southwestern Europe can be ascribed to the egg type Megaloolithidae, and more precisely to *Megaloolithus siruguei* and *Megaloolithus mamillare* egg-types. It is worthy comment that pathologic remains attributed to *M*. *siruguei* appear located in the uppermost section of its stratigraphic range, whiles those of *M*. *mamillare* occur in the lower part of its respective stratigraphic interval (Figs 1B and 4; see also Supplementary Information Table S1).

In France, the La Bégude site is considered the lowest stratigraphic locality yielding pathologic eggshell, which is dated as early Maastrichtian in age (within the lowermost part of the C31r magnetochron^30,36^). On the other hand, the Vitrolles-La Plain site is the stratigraphically uppermost pathologic-egg-bearing locality in the region, and dated as early late Maastrichtian (near the C31r-C31n reversal^37^). The lowest pathologic remains in the southern Pyrenees are found in the Mas de Pinyes-1 site, which falls in the lower part of the C31r, and therefore dated as early Maastrichtian^29,33^. On the other hand, the uppermost pathologic oological remains in the region come from the Els Nerets locality, falling in the middle part of the chron C31r^33^ and considered late early Maastrichtian in age.

We found highly statistical significant (x^2^ = 4.37 × 10^−17^; df = 6; p < 0.05; see Supplementary Information) the occurrence of pathologic remains in the lower Maastrichtian. A non-parametric confidence interval correction^38^ was also applied to establish the most parsimonious time-range for the pathologic remains. By taking the European record as a whole, the confidence interval factor was −0.019 (n = 7; p < 0.05), and the confidence time-interval expands from ~71.3 Ma to 70.1 Ma (Fig. 1B).

### Structure and stable isotope signal of abnormal titanosaurian eggshells

Most of analysed pathologic specimens come from egg debris (see Supplementary Information Table S2), which have been interpreted as disaggregated, or partially preserved but *in situ* eggs. A single complete egg exhibiting pathologic features was recovered from the Coll de Nargó nesting-area (Lleida province, Catalonia). The egg (MCD-5413) is ellipsoidal in shape, being the three semi-principal axes about X = 10.5 cm, Y = 8 cm, and Z = 5 cm, and its external surface is covered of irregular nodes. The ellipsoidal morphology of the egg is the result of tectonic compression linked to the Pyrenees formation rather than pathologic, given that all non-pathologic dinosaur eggs from Coll de Nargó exhibit similar morphology^28^.

**Figure 2:**
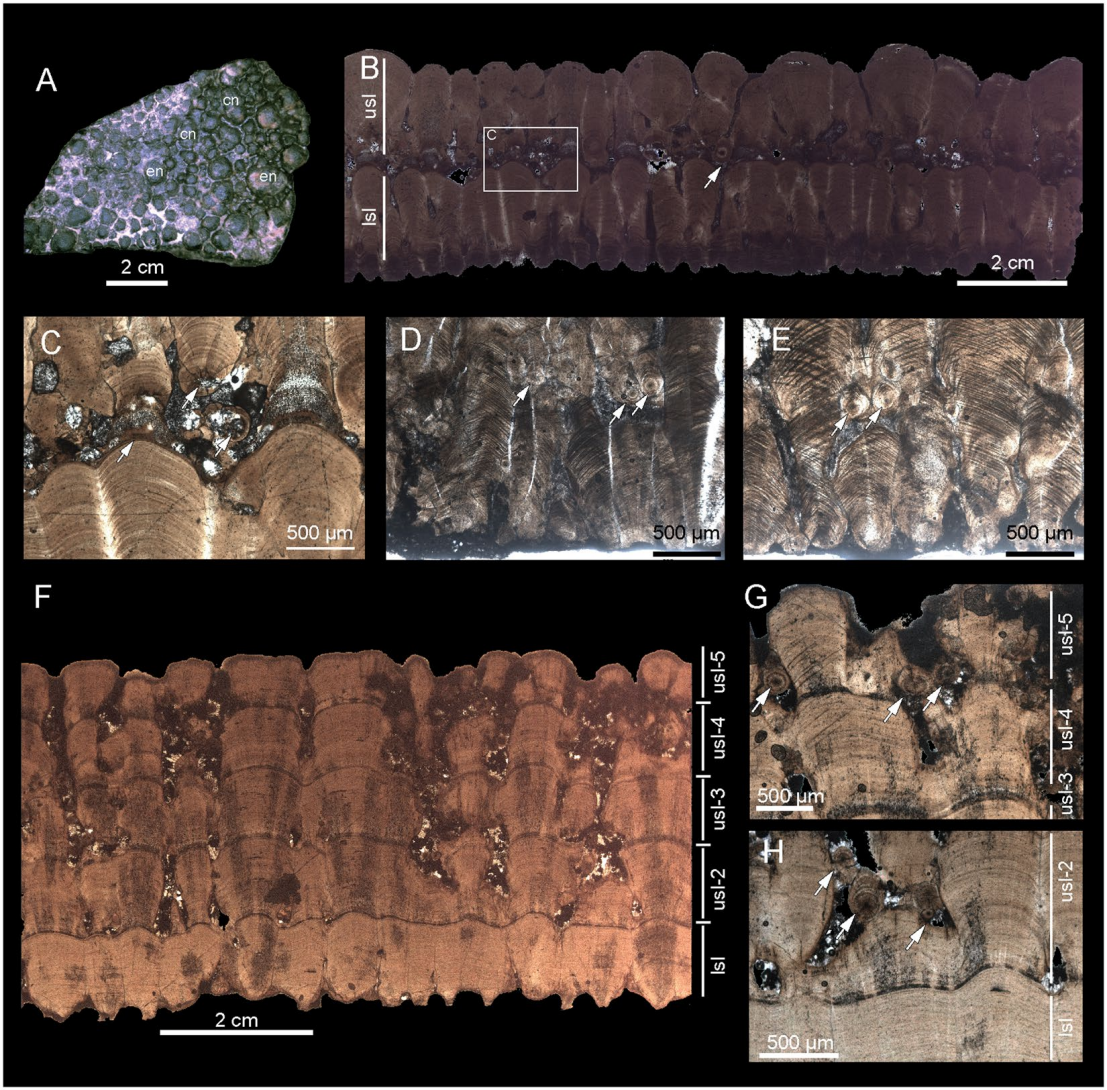
Pathologic characters. Pathologic features in *Megaloolithus siruguei* (**A**–**E**); IPS-59130 and IPS-59131) from the Coll de Nargó area, and *Megaloolithus mamillare* (**F**–**G**; IPS-59132) from the Els Nerets site. (**A**) External irregular surface of a pathologic *M*. *siruguei* eggshell; (**B**) general view of a radial thin section of a pathologic *M*. *siruguei* eggshell, and close up details (**C**–**E**) showing the distinct contact between superimposed eggshell layers and location of additional nucleation centres (white arrows). (**F**) Radial thin section of a “five-layered” pathologic *M*. *mamillare* eggshell, and close up details (**G**,**H**) showing the structural relation between superposed calcareous layers and location of additional nucleation centres (white arrows). Abbreviation: en, enlarged nodes; cn, coalescent nodes; lsl, lower shell layer; usl, upper shell layer.

Non-pathologic *M*. *siruguei* specimens are distinctively covered with rounded nodes of 0.5 to 1.23 mm in diameter^17,29^, while most of the pathologic eggshells exhibit aberrant ornamentation of irregular, enlarged (0.7 to 2.4 mm in diameter) and coalescent nodes (Fig. 2A). All the analysed pathologic specimens attributed to *M*. *siruguei* combine both superimposed eggshell layers and additional nucleation centres (Fig. 2B–E). A great disparity between the thicknesses of superimposed eggshell layers is observed (Supplementary Table S2), a condition that cannot be attributed to diagenetic alteration as occurs in other dinosaur egg-sites^18,39^. In some specimens the lower eggshell is well developed, showing a normal structure, while the upper one is thinner and shows more irregular shell units, as in some pathologic Argentinean sauropod eggs^13^. Extra nucleation centres occur at different points within pathologic eggshells, but mainly located at the basis of the overlaying eggshell, or near the boundary between superimposed eggshells. The boundary between the lower and the overlaying eggshell varies from well defined to irregular with vacuities frequently occupied by blocky sparry calcite and micrite cement. Because of that, no evidence of eggshell membrane is observed neither at the base of overlaying eggshells nor in the lower one.

The outer ornamentation of both normal and pathologic *M*. *mamillare* eggshells are nearly equal, being the size of nodes of 1 and 1.2 mm in diameter, respectively. The 5.35-mm-thick *M*. *mamillare* pathologic specimen from the Els Nerets locality (IPS-59132) is especially noteworthy because it consists of five superimposed eggshells layers (Fig. 2F). The normal lower eggshell layer is 1.35 mm in thickness, and covered by well-developed tubercular ornamentation. The overlaying abnormal eggshells are consecutively thinner than the previous ones (Supplementary Table S2), and calcite deposition follows the contour of the underlying eggshell, displaying smooth basal contacts (Fig. 2G,H). These structural features are equal to the Type III pathology described in Argentinean sauropod eggs^13^. The same pathological patterns are observed in other samples from the Els Nerets site (IPS-100376, IPS-100377), although they only show three superimposed eggshell layers. Similarly to pathologic *M*. *siruguei*, extra nuclear centres occur near the boundary of subsequent eggshell layers, but are especially abundant in the uppermost eggshell layer (Fig. 2G,H).

It is noteworthy that the occurrences of extra-spherulites have been used to infer pathologic conditions in dinosaur eggs^8,9^, but a recent study^39^ shows that those features occurring within primary shell units are crystallographic defects of taphonomic origin. However, this is not the case of the specimens presented herein, where extra nucleation centres and extra-spherulites mainly appears located between consecutive shell unit layers, thus reinforcing the suspicious of their pathologic origin.

**Figure 3:**
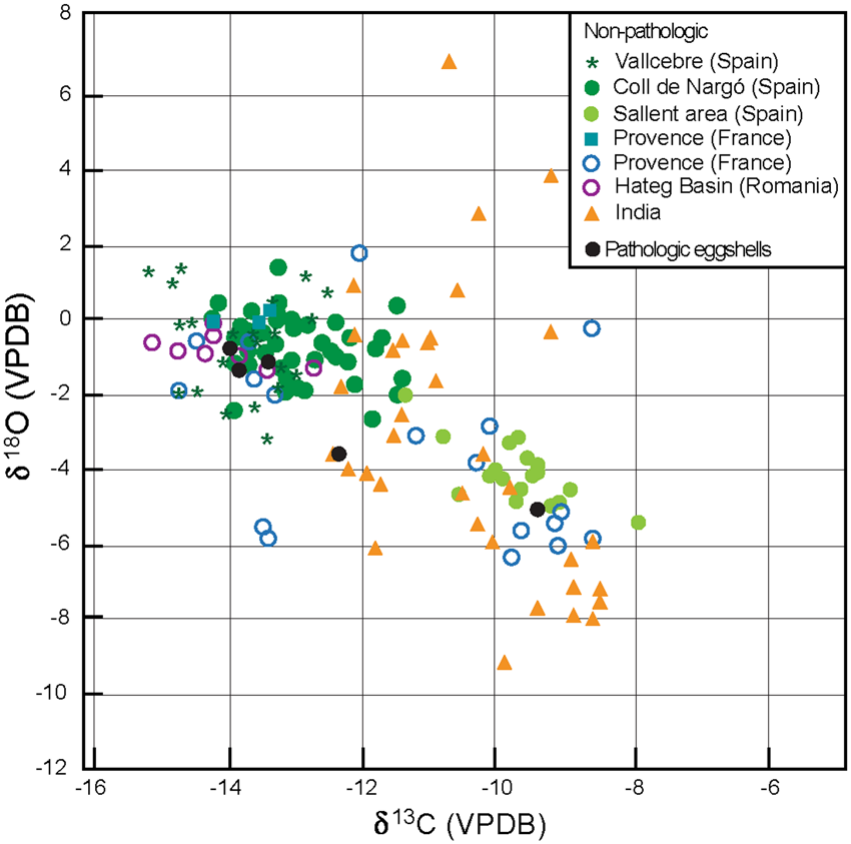
Isotopic signature. Plot of stable isotopic signature of both normal and pathologic titanosaurian egghells collected in Europe and other titanosaurian eggs worldwide^15,48,50,85,86^.

Isotopic signature of eggshells can provide significant information about the physiological conditions in which they were formed. Thus, if mechanisms causing the formation of pathologic eggshells were linked to some kind of physiological disorders, these anomalies should be reflected in a distinctive isotopic signature. According to our results, pathologic eggshells exhibit minor variations in δ^13^C and δ^18^O values, with δ^13^C values ranging between −9.3‰ and −13.85‰, and δ^18^O values between −0.80‰ and −5.11‰ (Supplementary Table S3). When data is represented as a δ^13^C/δ^18^O plot, all pathologic eggshells fall into the cluster of normal eggshells (Fig. 3).

## Discussion

Various authors^8,26,40–42^ have indicated that multi-layered eggshells or additional deposition of calcite in the shell of extant reptiles result from prolonged periods of egg retention within the female uterus, a phenomena known as dystocia. This retention of the egg by the female can be caused by reproductive stress induced by environmental, physiological, or ecological factors. The relatively short stratigraphic range of pathologic eggshell occurrences in the southwestern European basins suggest that they were produced as a consequence of a very particular event that mainly affected titanosaurian sauropods. In order to elucidate which factors could force titanosaurian females to retain their eggs, three scenarios are explored.

### Hypothesis 1, pathologic eggs result from an increase of reproductive stress by environmental changes

The hypothesis postulates that pathologies in titanosaurian eggs resulted from climatic perturbations that occurred at the end of the Cretaceous, as some authors already suggested more than 30 years ago^15,22–25^. According to this hypothesis climatic/environmental changes would have caused changes in their reproductive biology resulting in the production of abnormal eggs.

From a biological point of view, it is well known that certain physiologic and metabolic processes of reptiles are strongly temperature-dependent. Humidity and photoperiods are also important factors regulating the hormonal process involved in the reproductive cycle^43^. In extant reptiles, optimal environmental factors are essential for the reproductive health and immune system functioning. When environmental conditions exceed beyond the threshold of tolerance, many reptiles are obligated to suspend the development of the egg during the pre-ovipositional process^44,45^. As a consequence, several disorders may occur in both embryo and the egg.

Palaeoclimatic conditions of terrestrial environments can be inferred on the basis of the isotopic signal of pedogenic carbonates^46,47^. If drastic environmental shifts were the trigger event that led of the overproduction of pathologic eggs, these changes should be reflected as isotopic variations throughout stratigraphic sections^48,49^. According to the most recent isotopic data carried out in both northern Spain^50^ and southern France^48^, no significant climatic changes can be detected during the latest Cretaceous (Campanian-Maastrichtian) in southwestern Europe, at least in continental environments. Moreover, these studies point towards stable climatic conditions with low variation of the atmospheric temperature during the Maastrichtian, which agree with the global temperature fluctuation during that period^51^.

At the stratigraphic interval where pathologic eggshells occur (Fig. 4), no significant shifts in the δ^18^O or δ^13^C values are observed, and minor variations can be attributed to slight local fluctuations^48,50^. In the light of these data, there is not substantial evidence suggesting drastic climatic changes during the Maastrichtian in southwestern Europe, and hence it is no possible to establish any correlation between environmental perturbations and the occurrence of pathologic eggs.

### Hypothesis 2, pathologic eggs result from an increase of reproductive stress by changes in the dietary behaviour

Ewert and collaborators^26^ considered that possible changes in the feeding sources of sauropods would have produced physiological alterations affecting their reproductive cycle. Here we consider changes in diet such as those related with the resource quality or feeding behaviour, regardless of whether the later are linked or not with a major climatic change.

It is well known in the poultry industry that nutrition has a capital importance in the final egg quality and changes in the diet or a poor feeding source, especially those with inadequate proportion of calcium and vitamins, may have dramatic effect upon final egg structure^52^. Chemistry of drinking water (e.g. electrolyte imbalance or saline water) also might influence on the final egg quality^53,54^. For instance, a diet low in amino acid or poor in selenium may limit the egg production, whereas calcium-limited nutrition may prolong the laying period and causing binding eggs^55^.

In archosaurs, both crocodiles and birds, the calcareous layer of the egg is deposited in a separate uterine region of the reproductive tract^56^, resulting in a sequential shelling of the eggs; a condition also proved in theropod dinosaurs^57^. However, while in hens eggshell formation occurs just few hours before the egg laying, in alligators and crocodiles the egg takes longer than 24 hours^58^. In any case, given that calcium deposition occurs in a relatively short time, stable isotopic composition of the eggshell may reflect the nature of the last meals taken by the producer^59^. Thus, changes in the isotopic composition of the eggshell may reflect changes in feeding sources or dietary behaviour^49^.

The analysis of pathologic titanosaurian eggshells shows isotopic δ^13^C_egg_ and δ^18^O_egg_ values ranging from −9.3‰ to −13.85‰ and from −0.8‰ to −5.11‰, respectively. These values are similar to those reported in non-pathologic megaloolithid eggshells from the Maastrichtian of Iberia, southern France, Romania and India (Fig. 3). The isotopic signal of δ^13^C_egg_ in dinosaur eggshells is determined by the δ^13^C_diet_ value of the diet. Independently from the species, the eggshell is enriched in δ^13^C_egg_ about 16‰ relative to the ingested food^60,61^, and therefore, in herbivores, it may help in assessing the ingested vegetation. By considering this value of metabolic fractioning, the average δ^13^C_diet_ of the ingested vegetation by titanosaurians producing pathologic eggs fall between −25.3‰ and −29.85‰. By assuming similar fractioning rates for non-pathologic eggs coming from the same area^60^, the δ^13^C_diet_ of the ingested food is between −27.36‰ and −29.99‰. In both cases, values suggest an herbivorous diet based in C3 plants (average isotopic value of δ^13^C around −26‰, contra δ^13^C of −13‰ in C4 plants^62^) with very similar isotopic composition. Thus, it can be concluded that both normal and pathologic eggs were formed on the basis of similar feeding source.

The isotopic signal of δ^18^O_egg_ from eggshell informs on the nature of drinking water^48,63^, though the interpretation of its values is strongly subjected to the thermophysiology of the laying-taxon^15,63^. By assuming a virtual homeothermic condition for sauropods^64–66^, the δ^18^O values of their eggs can be directly related to the chemistry of the water they ingested. By considering the δ^18^O_egg_ values of studied pathologic eggshells, and using the equation correlating the δ^18^O of the eggshell and the δ^18^O of drinking water^67^, the resulting δ^18^O_water_ values of the ingested water for the analysed samples range between −2.71‰ and −9.55‰. These values are slightly lower compared to the average δ^18^O_water_ values inferred from non-pathologic eggshells reported from nearby areas of the southern Pyrenees (δ^18^O_water_ between −2.3‰ and −4.3‰), but similar to those observed in the Sallent site^50^ and the Provence area^15,48^. Because no differences in the δ^18^O_water_ values are recognized, we can conclude that both dinosaurs producing normal and pathologic eggshells probably drank water from similar sources. Finally, when the δ^13^C_egg_ and δ^18^O_egg_ values of normal and abnormal eggshells are plotted in a time-log chart, we observe that these are nearly invariable throughout the Maastrichtian section (Fig. 4), supporting the idea that there was not a significant change in the dietary behaviours, food type or quality of feeding resources of titanosaurian sauropods along the Maastrichtian.

### Hypothesis 3, pathologic eggs result from an increase of reproductive stress by ecological competition

The hypothesis explores the possibility that ecological competition among dinosaurs could produce reproductive stress and consequent dystocia. In extant reptiles, an increase of the inter- or intraspecific competition for resources or nesting area, such as in a faunal replacement scenario, may cause a strong reproductive stress, favouring the malfunction of the reproductive system and the production of abnormal eggs^68–70^.

From a physiologic perspective, it is demonstrated that stress, whatever its origin, alters the normal hormonal activity of the hypothalamus-pituitary-adrenocortical (HPA) axis in amniotes. Particularly noticeable are the modification on the baseline levels of the hypothalamic luteinizing hormone-releasing hormone (LHRH), the pituitaric follicle-stimulating hormone (FSH), the luteinizing hormone (LH) and prolactine, being all of them involved in the ovulation process^68–70^. In addition, this unbalanced hormonal cascade affects the levels of oestrogens participating in the metabolic regulation of the calcium for the formation of the eggshell^68–70^. The combination of both anomalous hormonal functions and inappropriate calcium deposition; whether due to excess or deficiency, may favour the occurrence of dystocia and the production of abnormal eggshells. Because titanosaurian sauropods could exhibit reptilian reproductive physiological traits^13^, similar physiological responses are expected in a similar scenario.

In southwestern Europe, a dinosaur faunal replacement is well documented at the end of the Cretaceous^71^. According to this major faunal change, the upper Campanian-lower Maastrichtian plant-eating dinosaurs, characterized by rhabdodontid ornithopods, nodosaurid ankylosaurs and titanosaurian sauropod taxa, were replaced by numerous new hadrosauroids and titanosaurian taxa. New taxa reached the Ibero-Armorican domain at some time around the early Maastrichtian-late Maastrichtian boundary. Furthermore, fossil evidence suggest that both associations coexisted for some time^71^. This faunal change can be also recognized on the basis of dinosaur ootaxa (Fig. 4). In such ecological scenario, it has been stated that the changing dinosaur community was subjected to a high ecologic stress^71^. Interestingly, the occurrence of abnormalities in titanosaurian eggs is stratigraphically correlated with that major turnover event (Fig. 4).

Because this turnover clearly affected multiple taxa, it seems plausible to suggest that during the period of cohabitation “old” and “new” dinosaur faunas could compete for similar, if not the same, ecologic resources in overlapped environments. For instance, titanosauran sauropods showed a general affinity for nesting in ancient soils developed upon floodplains^48,72–75^; and hadrosauroids also had an environmental preference for fluvial settings^76,77^. In southwestern Europe the environmental distribution of herbivorous dinosaurs concurs with this pattern, with nesting grounds of sauropods and habitat of hadrosauroids being reported primarily in fluvial settings^78–80^. This concurrence in certain environments could certainly have produced a significant increase of the ecological competition among different dinosaur groups for habitat preference and uses.

It still remains unclear the particular role that each group of dinosaurs played during this faunal replacement, but apparently the ecological perturbation would have distinctly affected upon each taxonomic group since titanosaurian sauropods are the only clade that suffered reproductive stress, while other taxa like rhabdodontids and nodosaurids become extinct in this process^71^.

## Methods

We compiled data from the megaloolithid egg localities reported in various basins of southwestern Europe, with special attention on the description and chronostratigraphic occurrence of pathological eggshells. In order to summarize the temporal occurrence of megaloolithid eggs in the entire region, we selected data from long stratigraphic successions of Provence area^17,30,48^, northern Pyrenees^30,32^, and southern Pyrenees^29,31,33,50,81^, which range from the upper Campanian to the uppermost Maastrichtian (Fig. 1, Supplementary Information Fig. S1). The stratigraphic position of ten egg levels yielding pathologic eggshells in France^16,17,30,82^ has been correlated with the most recent magnetostratigraphica data^32,37,48^ (Supplementary Information Fig. S2 and Table S1).

We sampled more than 90 consecutive stratigraphic egg-levels in 22 continuous and composite stratigraphic sections, of about 100 to 300 m thick each, distributed along the southern Pyrenees. For each egg-horizon, 50 eggshell samples were randomly collected from diverse both normal and abnormal *in situ* eggs or egg debris, taking only one sample per egg. As a result, we recovered 23 abnormal specimens in 14 egg-horizons along the southern Pyrenees (Fig. S1). A chronostratigraphic framework of the distribution of the Late Cretaceous dinosaur eggs from southern France (northern Pyrenees and Provence) was build up gathering information from the literature (see Supplementary Information for further details) and by first hand observations. As a result, 15 stratigraphic sections including 91 egg-horizons were correlated along this region (Fig. S2), but only 10 horizons have yield pathologic remains.

Eggshells preparation follows Val’s methodology^83^. Samples were preliminarily analysed using Leica® M60 binocular. Some eggshells were prepared as standard petrographic thin sections (30 µm), while others were examined and photographed using the environmental SEM Quanta 200 FEI, XTE 325/D8395 of the Scientific-technical Services of the University of Barcelona. Five pathologic eggshells (IPS-59122, IPS-59123, IPS-59124, IPS-59125, IPS-59127) were prepared for geochemical analysis using mass spectrometry. The isotopic analyses of dinosaur eggshells were performed at the Faculty of Geology of the University of Barcelona. The isotopic results are reported in per mil (‰) notation relative to the PeeDee Belemnite (VPDB) standard. The measured precision was over 0.1‰ for both the carbon and oxygen isotope compositions.

A chi-square test was performed in order to evaluate if there is, or not, a random stratigraphic distribution of pathologic dinosaur eggshells in southwestern Europe, assuming similar potential of fossilization for normal and pathologic eggshells, since both egg types were laid and incubated in the same fashion^28^. The chronostratigraphic log was divided in seven time-bins, from the upper Campanian to the late Maastrichtian (from 73 Ms to 66 Ma; Fig. 1), each one of 1 Ma (see Supplementary Information).

## Electronic supplementary material

Supplementary data
