# Supplementary material for: Evidence of Reproductive Stress in Titanosaurian Sauropods Triggered by an Increase in Ecological Competition
Source: Sci Rep. 2017 Oct 23;7:13827. doi: 10.1038/s41598-017-14255-6 (PMC5653779; doi:10.1038/s41598-017-14255-6)
Supplement: Supplementary file 1 — Supplementary data [file 41598_2017_14255_MOESM1_ESM.pdf]

EVIDENCE OF REPRODUCTIVE STRESS IN TITANOSAURIAN SAUROPODS TRIGGERED BY AN INCREASE IN ECOLOGICAL COMPETITION

ALBERT G. SELLÉS, BERNAT VILA, and ÀNGEL GALOBART

Table S1. List of uppermost Cretaceous sites from southwestern Europe yielding multi-layered pathologic eggshells.

Site (horizons)	Oospecies	Site reference	Age	Age reference
La Bégude (LB)	*M. siruguei*	17,31,82	early Maastrichtian	31
Vitrolles-Le Porry (VOP1)	*M. siruguei*	17,31,82	early Maastrichtian	31
Maupague-Carrefour (MC)	*M. siruguei*	17,31,82	early Maastrichtian	31
Rousset-Erben (ERR-1)	*M. mamillare*	17,31,82	early Maastrichtian	31
Ventabren (V)	*M. siruguei*	17,31,82	early Maastrichtian	31
La Cardeline (LC)	*M. mamillare*	17,28	early Maastrichtian	31
Font Mars (FM)	*Megaloolithus* indet.	82	early Maastrichtian	31
Roquehautes-Grand Creux (RGH-GG5b, RGH-GG8)	*M. siruguei*	17,31,28	late early Maastrichtian	31
Vitrolles-La Plaine (VLP-15)	*M. mamillare*	31,36	late early Maastrichtian	31,36
Sallent E	*M. siruguei*	This study	early Maastrichtian	29
Mas de Pinyes (Pinyes-A to G)	*M. siruguei*	This study	early Maastrichtian	34
Santa Eulàlia (Eulàlia A and B)	*M. siruguei*	This study	early Maastrichtian	29
Codó (Codó A and B)	*M. siruguei*	This study	early Maastrichtian	29
Els Nerets	*M. mamillare*	This study	late early Maastrichtian	31

Table S2. Main structural features of pathologic dinosaur eggshells from the southwestern Iberian Peninsula. Subscript numbers in abnormal layer(s) Thickness column indicate the position of the additional layers from the inner to the outer eggshell surface

Ref.	Oospecie	Source	Total thickness (mm)	Lower layer thickness (mm)	Upper layers thickness (mm)	Node diameter (mm)	Locality
IPS-59119a	*M. siruiguei*	egg debri	5.23	2.72	2.51	1.6-1.8	Sallent E
IPS-59119b	*M. siruiguei*	egg debri	5.30	2.80	2.50	2.2-2.3	Sallent E
IPS-59119c	*M. siruiguei*	partial egg	5.12	2.82	2.30	0.9-1.0	Sallent E
IPS-59119d	*M. siruiguei*	egg debri	5.24	2.62	1.62	1.8-2.0	Sallent E
IPS-59120a	*M. siruiguei*	egg debri	4.51	2.01	2.50	1.6-1.8	Pinyes-A
IPS-59120b	*M. siruiguei*	egg debri	4.61	1.05	3.55	0.7-1.1	Pinyes-A
IPS-59121	*M. siruiguei*	partial egg	5.25	2.84	2.41	2.2-2.4	Pinyes-B
IPS-59122	*M. siruiguei*	egg debri	3.98	1.52	1.45	0.6-1.1	Pinyes-C
MCD-5413	*M. siruiguei*	complete egg	3.74	1.89	1.85	1.0-1.3	Pinyes-C’
IPS-59123	*M. siruiguei*	egg debri	4.15	1.35	2.80	0.8-1.3	Codó-A
IPS-59124	*M. siruiguei*	partial egg	4.25	2.50	1.75	1.0-1.2	Pinyes-D
IPS-59125	*M. siruiguei*	egg debri	3.30	1.7	1.60	1.1-1.4	Pinyes-D
IPS-59126	*M. siruiguei*	partial egg	4.30	2.7	1.5	0.5-0.8	Codó-B
IPS-59127	*M. siruiguei*	egg debri	2.75	1.65	1.10	1.1-2.1	Codó-B
IPS-59128	*M. siruiguei*	egg debri	3.22	1.60	1.62	0.7-0.9	Eulàlia-A
IPS-59129	*M. siruiguei*	egg debri	3.30	1.75	1.55	1.1-1.5	Eulàlia-B
IPS-59130	*M. siruiguei*	egg debri	4.76	2.31	2.45	1.1-1.4	Pinyes-D
IPS-59131	*M. siruiguei*	egg debri	3.98	1.12	2.86	0.8-1.2	Pinyes-E
IPS-59132	*M. mamillare*	egg debri	5.30	1.35	$1.20^2/1.12^3/0.91^4/0.73^5$	1.0-1.2	Els Nerets
IPS-89571	*M. siruiguei*	egg debri	4.16	2.18	1.98	0.7-1.1	Pinyes-F
IPS-89572	*M. siruiguei*	egg debri	3.55	1.74	1.81	1.2-1.4	Pinyes-G

IPS-100376	*M. mamillare*	egg debri	3.69	1.84	1.02[2]/0.83[3]	1.1-1.2	Els Nerets
IPS-100377	*M. mamillare*	egg debri	3.81	1.78	0.83[2]/1.20[3]	1.0-1.3	Els Nerets
normal M. siruguei		compete egg	1.75-3.63	-		0.5-1.2	Coll de Nargó
normal M. mamillare		compete egg	1.20-2.25	-		0.3-1.2	Els Nerets

Table S3. Stable isotopic values δ ^{13}C and δ ^{18}O from pathologic dinosaur eggshells from the southwestern Iberian Peninsula. Abbreviations: PDB, Pee Dee Belemnite; SMOW, Standard Mean Ocean Water.

Sample	‰ δ^{13}C (PDB)	‰ δ^{18}O (PDB)	‰ δ^{18}O (SMOW)
IPS-59122	-12,24	-3,62	27,1
IPS-59123	-13,29	-1,06	29,8
IPS-59124	-13,85	-0,80	30,0
IPS-59125	-9,30	-5,11	25,6
IPS-59127	-13,74	-1,28	29,5

Table S4. Data used for the chi-square analysis testing the non-random stratigraphic distribution of pathologic eggs in southeastern Europe.

Time interval (in Ma)	Nº of egg-horizons	Nº of samples per time bin	Expected nº of pathologic eggs (assumption at 2%)	Real nº of pathologic eggs
66-67	9	450	9	0
67-68	1	50	1	0
68-69	1	50	1	0
69-70	23	1150	23	0
70-71	88	3450	69	19
71-72	30	1250	25	5
72-73	3	150	3	0

Figure Captions

Figure S1. Magnetostratigraphic panel of the dinosaur egg-bearing levels from the latest Cretaceous of Southern Pyrenees. Egg-horizons with associated repository numbers indicates the precedence of pathologic eggs samples.

Stratigraphic data [34,78,81,84]; Magnetostratigraphic data [34,78,81,84]; Dinosaur egg-horizon location [29,32,34,78,81,84].

Figure S2. Magnetostratigraphic panel (no at scale) of the dinosaur egg-bearing levels from the latest Cretaceous of Southern France. Pathologic egg-bearing levels are indicated as: ERR-1, Rousset-Erben; FM, Font Mars; LB, La Bégude; LC, La Cardeline; MC, Maupage-Carrefour; RGH-GG5b and RGH-GG8, Roquehautes; V, Ventabren; VLP-15, Vitrolles-La Plaine; VPO1, Vitrolles-Le Porry. Stratigraphic data [31]; Magnetostratigraphic data [31,33,36,48,S1,S2,S3,S4]; Dinosaur egg-horizon location [17,31,33,36,48,82,S1,S5].

Additional References

S1. Cojan, I. & Moreau, M.-G. Correaltion of terrestrial climatic fluctuation with global signal during the upper Cretaceous-Daninan in a compressive setting (Provence, France). *Jour. Sedim. Res*. **76**, 589—604 (2006).

S2. Galbrun, B. Did the European dinosaurs disappear before the K-T event? Magnetostratigraphic evidence. *Earth. Plan. Sci. Lett*. **148**, 569—579 (1997).

S3. Benammi, M., Urrutia-Fucugauchi, J. & Vianey-Liaud, M. Preliminary magnetostratigraphic study of the Late Cretaceous dinosaur site from Villeveyrac-Mèze Basin, Southern France. *Int. Geo. Rev*. **48**, 89—96 (2006).

S4. Krumsiek, K. & Hahn, G. G. Magnetostratigraphy near the Cretaceous-

Tertiary Boundary near Aix-en-Provence (Southern France). Cahi. Rév. Géol. Prov. (Digne, 1989).

S5. García, G., Feist, M., Cabot, A., Valentin, X. & Vianey-Liaud, M. Les oeufs de dinosaur du Crétacé supériue du basin de Villeveyrac-Mèze (Hérault, France): description d'une novella ooespèce de *Prismatoolithus. Soc. Geol. Frances* **171**, 283-289 (2000).

Figure S1

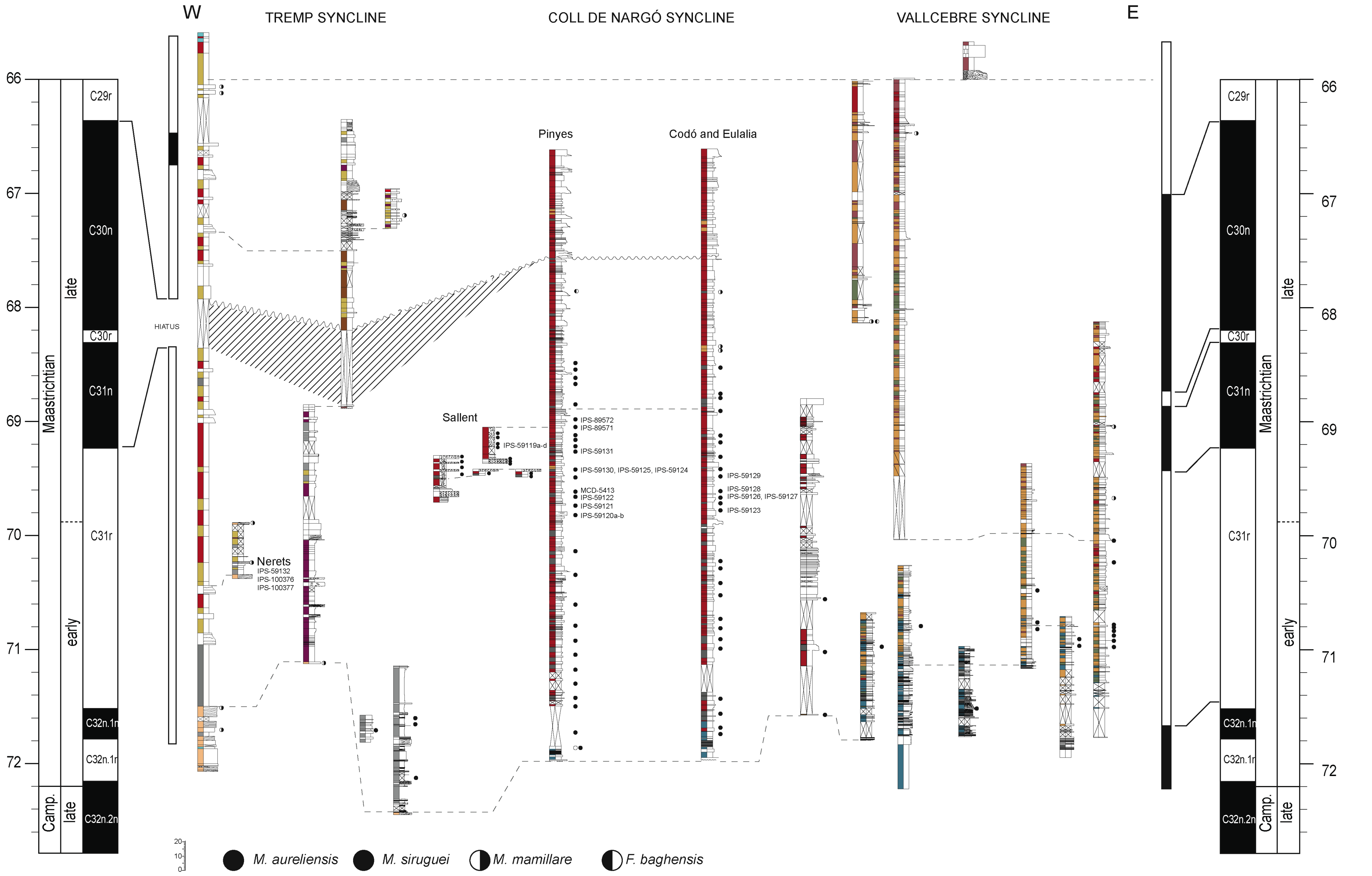
W
TREMP SYNCLINE
COLL DE NARGÓ SYNCLINE
VALLCEBRE SYNCLINE
E
66
67
68
69
70
71
72
Maastrichtian
Camp.
late
early
C29r
C30n
C30r
C31n
C31r
C32n.1n
C32n.1r
C32n.2n
HIATUS
Pinyes
Codó and Eulalia
Sallent
Nerets
IPS-59132
IPS-100376
IPS-100377
IPS-59119a-d
IPS-89572
IPS-89571
IPS-59131
IPS-59130, IPS-59125, IPS-59124
MCD-5413
IPS-59122
IPS-59121
IPS-59120a-b
IPS-59129
IPS-59128
IPS-59126, IPS-59127
IPS-59123
20
10
0
M. aureliensis
M. siruguei
M. mamillare
F. baghensis

Figure S2

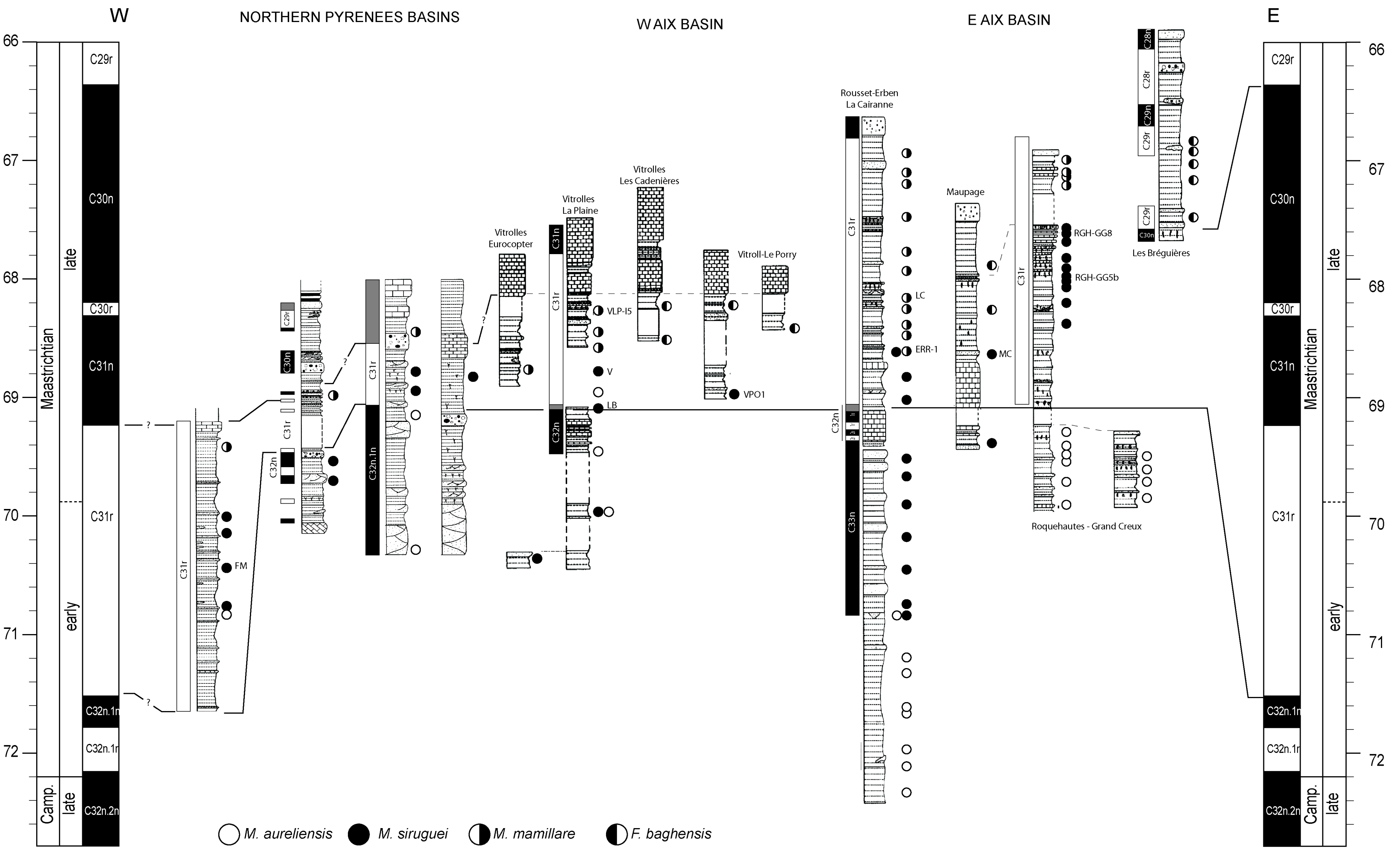

W
NORTHERN PYRENEES BASINS
W AIX BASIN
E AIX BASIN
E
Maastrichtian
late
early
Camp.
C29r
C30n
C30r
C31n
C31r
C32n.1n
C32n.1r
C32n.2n
C32n
C33n
C28r
C28n
C29n
Vitrolles Eurocopter
Vitrolles La Plaine
Vitrolles Les Cadenières
Vitroll-Le Porry
Rousset-Erben La Cairanne
Maupage
Les Bréguières
Roquehautes - Grand Creux
FM
VLP-I5
V
LB
VPO1
LC
ERR-1
MC
RGH-GG8
RGH-GG5b
M. aureliensis
M. siruguei
M. mamillare
F. baghensis
